# Supplementary material for: Diffusion MRI measurements in challenging head and brain regions via cross-term spatiotemporally encoding
Source: Sci Rep. 2017 Dec 21;7:18010. doi: 10.1038/s41598-017-17947-1 (PMC5740132; doi:10.1038/s41598-017-17947-1)
Supplement: Supplementary file 1 — Supporting Information [file 41598_2017_17947_MOESM1_ESM.pdf]

Supporting Information for

Diffusion MRI measurements in challenging head and brain regions via cross-term spatiotemporally encoding

Eddy Solomon[a], Gilad Liberman[a], Zhiyong Zhang and Lucio Frydman*

Department of Chemical and Biological Physics, Weizmann Institute of Science, Rehovot 76100, Israel

A. Theoretical analysis of diffusion in xSPEN MRI.

To provide a quantitative framework capable of estimating the effects that diffusion will have in xSPEN MRI, we rely on a formalism whereby the local derivatives of the spin evolution phases are first calculated, and then combined with the diffusion model discussed by Karlicek and Lowe (1) according to which the diffusion-derived attenuation imposed by gradients throughout an NMR sequence is summarized by a *b*-value

$$b(t) = \gamma^2 \cdot \int_0^t K^2(t')\,dt' \qquad \text{[S1]}$$

where $K(t') = \int_0^{t'} G(t'')\,dt''$ is a wavenumber encompassing the action of all gradients up to a particular time *t'*. As shown in (2,3), the application of frequency swept pulses under the action of gradients as done in SPEN/xSPEN, results in a spin dephasing which, by contrast to the assumptions leading to Eq. [S1], is neither linear in space, nor independent of position. To account for this we preserve Eq. [S1] but re-express the *K*-wavenumber in terms of a local spatial dispersion K^{local}, describing the dephasing experienced by the spins within a neighboring region that is relevant in terms of the diffusion length scale (2,4). A Taylor expansion allows one to describe this wavenumber in proximity to an arbitrary r_0 as $K^{local}(t',r_o) = \frac{d\varphi(t',r_o)}{dr}$, an expression that becomes identical to the Karlicek-Lowe formulation if φ's dephasing has been imparted solely by a linear gradient.

Using this formalism for calculating the diffusion-driven signal attenuation as a function of time and position, the decays expected for the xSPEN sequences described in Figure 1 of the main text, were estimated. For simplicity, we only took into consideration the imaging gradients

along the xSPEN-relevant (y,z)-axes, disregarding the effects of the rapidly-oscillating RO (x-axis) gradient, and assuming that the diffusion gradients (greyed G_ds in Fig. 1) were initially null. The relevant manipulations therefore include a slice-selective 90° excitation followed by two identical frequency-swept inversion pulses acting in synchrony with a bipolar $\pm G_y$ –all of this imparted while in the presence a constant G_z, which stays active throughout the course of an acquisition lasting a duration T_a. Referring to t_- as the time in which spins positioned at a given $r_o=(y_o,z_o)$ coordinate are addressed by the first inversion pulse, t_+ as the time when these spins are addressed by the second pulse, and $T_e=T_a/2$ as the duration of these pulses, the formalism described in Refs (2,5) allows one to compute the local wavenumbers accumulated by the spins during the course of the xSPEN encoding and up to the instant of their subsequent acquisition as

$$K^{local}(t',r_o)=\begin{cases} -G_y t+G_z t, & 0\le t\le t_- \\ G_y t-G_z t, & t_-\le t\le T_e \\ G_y t-G_z t, & T_e\le t\le T_e+\dfrac{T_a}{2} \\ \dfrac{G_y}{2}\left(-2t+T_a+4T_e\right)-G_z t, & T_e+\dfrac{T_a}{2}\le t\le T_e+\dfrac{T_a}{2}+t_+ \\ G_y t-\dfrac{G_y T_a}{2}+G_z(t-T_a-2T_e), & T_e+\dfrac{T_a}{2}+t_+\le t\le T_e+\dfrac{T_a}{2}+T_e \\ 2G_y T_e+G_z t-\dfrac{G_z T_a}{2}, & 0\le t\le T_a \end{cases} \qquad \text{[S2]}$$

Using such dynamic evolution wavenumbers, the b-values can be calculated from Eq. [S1] in a similar piece-wise temporal fashion as

$$b(t)=\gamma^2\begin{cases} \dfrac{\left(G_y^2+G_z^2\right)t_-^3}{3}, & 0\le t\le t_- \\ -\dfrac{\left(G_y^2+G_z^2\right)\left(t_-^3-T_e^3\right)}{3}, & t_-\le t\le T_e \\ \dfrac{T_a\left(12G_y^2T_e^2+G_z^2\left(T_a^2+6T_aT_e+12T_e^2\right)\right)}{24} & T_e\le t\le T_e+\dfrac{T_a}{2} \\ \dfrac{t_+\left[4G_y^2\left(t_+^2-3t_+T_e+3T_e^2\right)+G_z^2\left(3T_a^2+6T_a\left(t_++2T_e\right)+4\left(t_+^2+3t_+T_e+3T_e^2\right)\right)\right]}{12} & T_e+\dfrac{T_a}{2}\le t\le T_e+\dfrac{T_a}{2}+t_+ \\ -\dfrac{G_y^2t_+^3}{3}-G_y^2t_+^2T_e-G_y^2t_+T_e^2+\dfrac{7G_y^2T_e^3}{3}+\dfrac{G_z^2\left(-T_a^3+\left(T_a-2t_++2T_e\right)^3\right)}{24} & T_e+\dfrac{T_a}{2}+t_+\le t\le T_e+\dfrac{T_a}{2}+T_e \\ \dfrac{G_z^2T_a^3}{12}+4G_y^2T_aT_e^2 & 0\le t\le T_a \end{cases} \qquad \text{[S3]}$$

From this equation the diffusion-driven decay $S(b)/S(0)=\exp[-b{\cdot}D]$ affecting xSPEN even in the absence of diffusion-sensitizing gradients –where D is an isotropic diffusion coefficient and $S(0)$ is the signal in the absence of diffusion– follows.

In general, displacement measurements require extending the above calculations to account for the presence of diffusion gradients G_d applied along multiple, non-coincident spatial orientations (6-8). These gradients can be introduced in the xSPEN scheme as illustrated in the manuscript's Fig. 1a, which places a PGSE block during a built-in $(T_a+p_1)/2$ free evolution delay introduced for the sake of achieving full refocusing. These pulsed magnetic field gradients G_d, acting along arbitrary orientations for a duration δ and separated by a diffusion-sensitizing time Δ, can be accounted by extending the K^{local}-based formalism that lead to Eq. [S2] to the tensorial form (9)

$$\bar{\bar{b}}(t,r_0) = \gamma^2 \int_0^t (\vec{\nabla}(\varphi(t',r_0))\vec{\nabla}(\varphi',r_0)^{\mathrm{T}})dt', \qquad \text{[S4]}$$

where $\bar{\bar{b}}$ is now a matrix dictated by products of the localized phase derivatives of $\varphi(t',r_0)$, given by both the imaging and the diffusion gradients. The resulting $\bar{\bar{b}}$ tensor will be both space- and time-dependent, and by contrast to common PGSE measurements, it will have usually two dominant eigenvalues that define it. Complexities of this behavior are further illustrated in Extended Data Figure S1, which describes how the various tensorial *b*-components vary for different positions along the imaged axis, over the course of an xSPEN acquisition.

B. Gradient scheme validations.

The reliability of the various gradient schemes discussed in the main text's Figure 2 were tested on a set of 2601 "synthetic tissues", where each of these samples was assigned a random proton density and axially-symmetric diffusion tensors with randomized directionality and eigenvalues spanning realistic FA (0-1 arbitrary) and ADC ($0.4\text{-}1.8\text{x}10^{-3}$) values. The signals arising from these different tissues under the action of the various gradient schemes were simulated using the Bloch-Torrey formalism (10), and Gaussian noise was added to these calculated signals so that the mean b_0–image SNR would be 7%. Based on these synthetic sets, FA and ADC values were then estimated, and both mean absolute differences (which can be appreciated in Fig. 2 as deviations from the red-lined unity slopes graphed) and r^2 values, were calculated against the ground truth FAs and ADCs for the various tested gradient schemes. The Extended Data Figure S2 illustrates an experimental validation of the resulting b_o-including "double-cone" approach, conducted on a water phantom in a clinical 3T MRI machine, showing essentially the same

reliability as EPI-based maps. This latter gradient scheme was adopted for the human ADC and DTI xSPEN mapping.

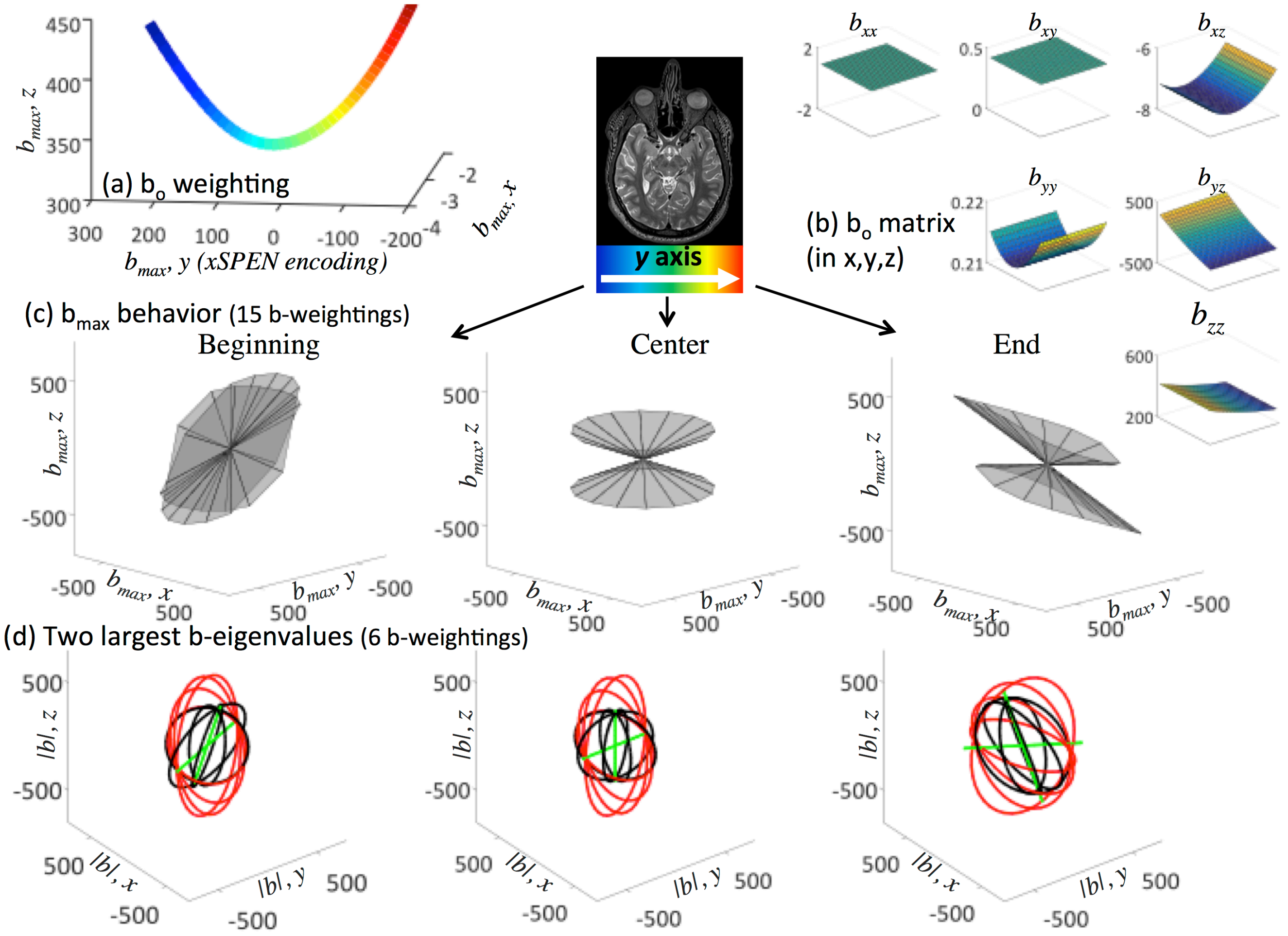

Extended Data - Figure S1. Features associated to xSPEN's use in diffusion measurements, illustrated with results computed on the basis of Eqs. [S1]-[S4]. For all axes in all panels, values are given in units of s/mm^2. Simulation parameters were taken from the clinical scanner experiments (see Methods). (a) Curve connecting the tips of the largest $\bar{\bar{b}}$-matrix eigenvector (b_{max}) arising in xSPEN in the absence of diffusion-sensitizing gradients; the b-components in this b_o experiment are shown projected onto the (x,y,z) imaging axes. Colors correspond to progress in the xSPEN image decoding; i.e., along the white arrow shown under the brain image. In correspondence with the acquisition parameters used for the experiments in the main text's Fig. 4, the spatial dependency of these b-values is strong and variable along the y-axis, sizable and nearly constant along the z-axis (~400 s/mm^2), and negligible along the x-axis. (b) Complementary rendering of this $\bar{\bar{b}}$-tensor information, showing the different weightings exhibited by the $\bar{\bar{b}}_o$-matrix components in the (x,y,z) space. (c) Description of how the largest b-tensor element varies over the course of xSPEN acquisitions involving an array of 15 PGSE gradients rotating in the (x,y)-plane, showing the position-dependent distortions introduced by the xSPEN imaging

sequence. (d) Coverage of the $\overline{\overline{b}}$-matrix components afforded by the double-cone gradient scheme (Fig 2c; only six G_d values chosen for a clearer presentation) focusing on the "coin-like" structures subtended by $\overline{\overline{b}}$'s two largest-valued eigenvectors. These disks are shown as a function of *y*-position when choosing the SS axis along *z* (black ellipses) or along *x* (red ellipses). The green axes represent the two b_o samples associated with these orthogonal choices.

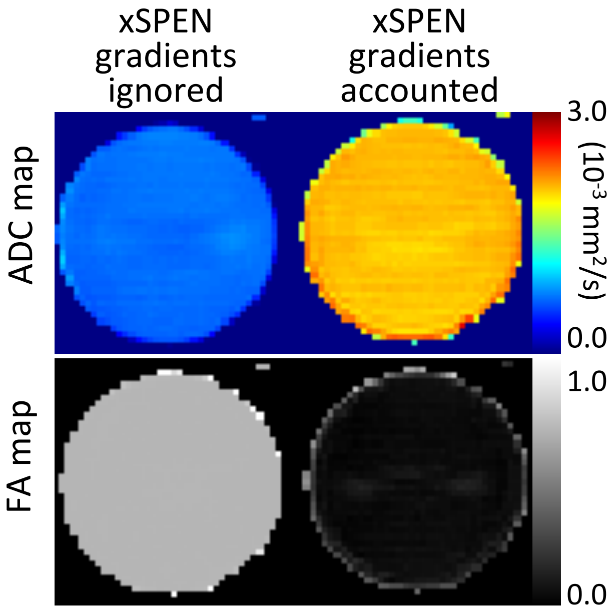

Extended Data Figure S2. Experimental validation of the "b_o-enhanced double-cone" gradient scheme introduced in the main text Figs. 1b and 2d, on a water phantom examined in a 3T clinical scanner. The xSPEN pulse sequence in Fig. 1b was used to derive the ADC and FA maps. In the left-hand column these were derived under the assumption that the diffusion-driven signal attenuation solely arise due to the effects of the G_d diffusion gradients (11); in the right-hand column maps accounted for both the xSPEN imaging and the PGSE bipolar gradients as per the analytical calculation deriving from Eqs. [S1]-[S4].

References

1. Karlicek RF, Lowe IJ. Modified Pulsed Gradient Technique for Measuring Diffusion in the Presence of Large Background Gradients. J Magn Reson 1980;37(1):75-91.
2. Shrot Y, Frydman L. The effects of molecular diffusion in ultrafast two-dimensional nuclear magnetic resonance. J Chem Phys 2008;128(16):164513.
3. Tal A, Frydman L. Single-scan multidimensional magnetic resonance. Prog Nucl Magn Reson Spectrosc 2010;57(3):241-292.
4. Shrot Y, Frydman L. Single-scan 2D DOSY NMR spectroscopy. J Magn Reson 2008;195(2):226-231.
5. Solomon E, Shemesh N, Frydman L. Diffusion weighted MRI by spatiotemporal encoding: analytical description and in vivo validations. J Magn Reson 2013;232:76-86.

6. Mattiello J, Basser PJ, Lebihan D. Analytical Expressions for the B-Matrix in Nmr Diffusion Imaging and Spectroscopy (Vol 108, Pg 131, 1994). J Magn Reson Series A 1994;111(2):232-232.
7. Price WS. Pulsed-field gradient nuclear magnetic resonance as a tool for studying translational diffusion: Part 1. Basic theory. Concepts in Magnetic Resonance Part A 1997;9(5):299-336.
8. Price WS. Pulsed-field gradient nuclear magnetic resonance as a tool for studying translational diffusion: Part II. Experimental aspects. Concepts in Magnetic Resonance Part A 1998;10(4):197-237.
9. Solomon E, Liberman G, Nissan N, Frydman L. Robust diffusion tensor imaging by spatiotemporal encoding: Principles and in vivo demonstrations. Magn Reson Med 2017;77(3):1124-1133.
10. Torrey HC. Bloch Equations with Diffusion Terms. Phys Rev 1956;104(3):563-565.
11. Stejskal EO, Tanner JE. Spin Diffusion Measurements: Spin Echoes in the Presence of a Time-Dependent Field Gradient. Journal of Chemical Physics 1965;42(1):288-292.
